# A Sarcomatoid Renal Cell Carcinoma with Clear Cell Papillary-Like Primary Tumor and Lymph Node Metastasis: A Diagnostic Conundrum

**DOI:** 10.1177/10668969221145011

**Published:** 2022-12-22

**Authors:** Nadine Demko, Kate I Glennon, Madeleine Arseneault, Katherine Lach, Tamiko Nishimura, Simon Tanguay, Yasser Riazalhosseini, Fadi Brimo

**Affiliations:** 1 Department of Anatomical Pathology, 5620 McGill University, Montréal, Québec, Canada; 2 Department of Human Genetics, 5620 McGill University, Montréal, Québec, Canada; 3 McGill Genome Centre, 5620 McGill University, Montréal, Québec, Canada; 4 Division of Urology, 5620 McGill University, Montréal, Québec, Canada

**Keywords:** clear cell papillary renal cell tumor, sarcomatoid renal cell carcinoma, lymph node metastasis, immunohistochemistry, fluorescence in situ hybridization, next-generation sequencing, whole exome sequencing, copy number variation

## Abstract

Clear cell papillary renal cell tumor (CCPRCT) is a distinct clinical entity with characteristic pathological features and non-aggressive clinical behavior. Diagnostically challenging cases present when there are immunomorphological findings of CCPRCT associated with heterogeneous morphologies, aggressive histological features, and advanced pathological stages—so-called CCPRCT-like tumors. In this report, we describe a heterogeneous, multifocal renal tumor with immunomorphological characteristics of CCPRCT but with associated aggressive features such as sarcomatoid and necrotic areas, perirenal and sinus fat involvement, and most notably, lymph node metastasis composed entirely of classic CCPRCT morphology and immunophenotype. Immunohistochemical and fluorescence *in situ* hybridization studies did not support a translocation renal cell carcinoma. Molecular analyses did not identify common mutations or chromosomal abnormalities seen in clear cell renal cell carcinoma or *ELOC*-mutated renal cell carcinoma. This case highlights that rare renal cell tumors remain difficult to classify and the distinction between CCPRCT and CCPRCT-like tumors remains to be better defined.

## Case Report

An 83-year-old female was incidentally found to have a renal mass and retroperitoneal adenopathy on imaging. Her medical history was significant for a transurethral resection of a noninvasive low-grade papillary urothelial carcinoma. Computed tomography (CT) scan revealed a predominantly solid, 3.2 cm mass with cystic areas in the left kidney with a predominantly cystic, 4.0 cm left periaortic mass. Renal biopsy identified an unclassifiable renal cell carcinoma (RCC) with sarcomatoid differentiation. Lymph node biopsy identified minute fragments of tubulopapillary structures suggestive of metastatic RCC. Preoperative bone scan and CT thorax did not show bone or pulmonary metastases. The patient was scheduled for a left open radical nephrectomy and lymph node dissection.

**Figure 1.**
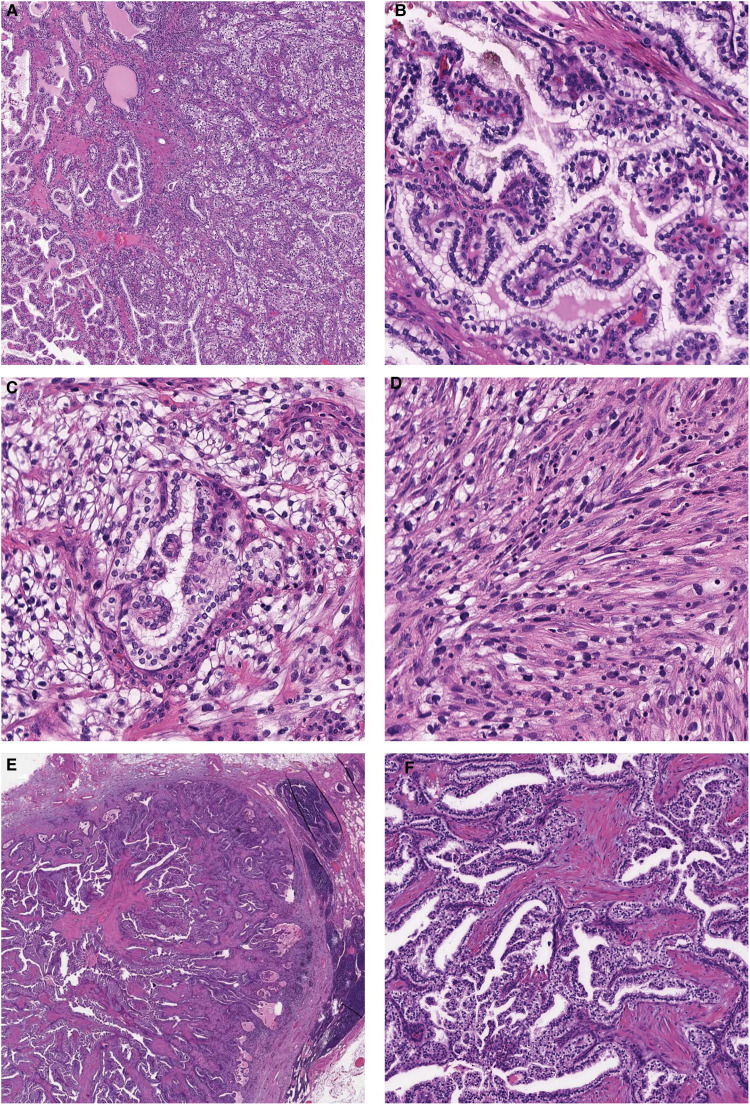
H&E light microscopic morphology of the primary tumor and lymph node metastatic focus. (A) Low-power view of tumor 1 showing classic CCPRCT morphology on the left with more solid nested morphology on the right. (B) Intermediate magnification of the CCPRCT morphology illustrating classic tubulopapillary architecture of CCPRCT. Intermediate power of the areas with (C) solid nested architecture and (D) sarcomatoid transformation, both aberrant CCPRCT morphologies identified in tumor 1. (E) Low-power view of the lymph node metastasis showing the CCPRCT focus with a lymphoid rim. (F) Intermediate magnification of the classic CCPRCT morphology seen in the lymph node metastatic focus. Abbreviations: CCPRCT, clear cell papillary renal cell tumor; H&E, hematoxylin and eosin.

**Figure 2.**
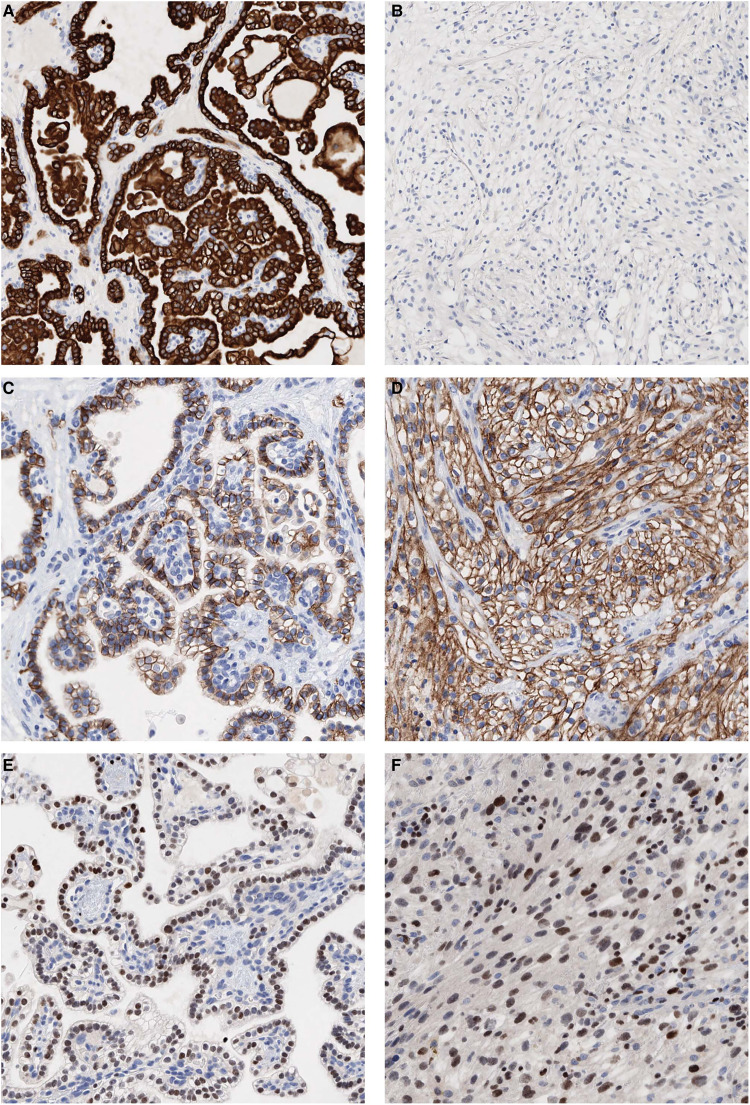
Immunohistochemical staining of the CCPRCT primary tumor from both the classic component as well as the solid nested, sarcomatoid areas. (A) Strong positive staining for keratin 7 in the classic CCPRCT component and (B) loss of expression in the solid nested areas. (C) Strong positive “cup-like” staining for CA9 in the classic component and (D) membranous staining in the solid nested areas. (E) GATA3 moderate to strong staining in the classic CCPRCT component and (F) moderate to strong staining in the sarcomatoid areas. Abbreviations: CA9, carbonic anhydrase 9; CCPRCT, clear cell papillary renal cell tumor; GATA3, GATA-binding protein 3.

Macroscopic examination of the left nephrectomy specimen identified two tumors: the first (tumor 1) measured 3.0 × 2.7 × 2.5 cm and the second (tumor 2) measured 3.7 × 2.4 × 2.0 cm. Tumor 1 showed histological features typical of clear cell papillary renal cell tumor (CCPRCT), merging with areas displaying more compact cellular growth as well as spindle cell sarcomatoid differentiation. Immunohistochemistry revealed the following staining pattern in the clear cell papillary component: diffuse strong staining for PAX8, keratin AE1/AE3, keratin 8/18, keratin 7, GATA3, carbonic anhydrase 9 (CA9) in a “cup-like” fashion, apical staining for CD10, and negative staining for AMACR. The sarcomatoid component was diffusely positive for CA9, CD10, and GATA3 and negative for AMACR and keratin 7. Fumarate hydratase (FH) and succinate dehydrogenase (SDH) were both retained, thereby ruling out FH-deficient and SDH-deficient RCCs. TFE3, cathepsin-K, MART-1, and HMB45 were also performed to rule out microphthalmia transcription factor family translocation RCCs and were negative. Confirmatory fluorescence *in situ* hybridization (FISH) with dual color break apart probe for *TFE3* did not identify *TFE3* gene rearrangements, thus eliminating *TFE3*-rearranged RCC. Given the morphological and immunohistochemical findings, tumor 1 was diagnosed as RCC with clear cell papillary and sarcomatoid features (see Figures 1 and 2). This tumor also showed areas of necrosis as well as invasion of the perirenal and renal sinus adipose tissue. Tumor 2 was organ-confined and had classic features of CCPRCT with a cystic component. Surprisingly, one lymph node out of five resected nodes contained metastatic RCC with a morphology (Figure 1E and F) and immunohistochemical profile consistent with CCPRCT. The final pathological stage was pT3a pN1.

Due to the highly unusual finding of a CCPRCT with sarcomatoid features and advanced pathological stage, further studies were needed to exclude other renal tumors that could show significant overlapping features with CCPRCT, such as clear cell RCC (CCRCC) and *ELOC* (formerly *TCEB1*)-mutated RCC. In order to do so, we interrogated the DNA sequences of different tumor components (primary CCPRCT-like component, primary sarcomatoid RCC, metastatic RCC), using an RCC-focused next-generation sequencing (NGS) gene panel^1^ followed by whole exome sequencing on an iSeq 100 and NovaSeq, respectively (paired-end 150). Bioinformatic analysis to detect germline and somatic variants was conducted as previously described.^1^ The analysis did not detect any mutations in the most commonly mutated genes in RCC, including *VHL, ELOC, PBRM1, SETD2, BAP1, MET, ATM, ARID1A, ATP9B, CDKN2A, CDKN2B, COL11A1, CSMD3, DMD, KDM5C, KDM6A, MTOR, NF2, NFE2L3, PTK7, PTEN, PIK3CA, TP53, TRRAP, TSC1*, and *TSC2* in any of the tumor components. Furthermore, copy number variation analysis did not detect loss of the *VHL* gene or whole chromosome losses in any of the areas examined.

Five months post resection, the patient was found to have a solitary lytic lesion in the vertebral body of L2 suspicious for metastasis that measured 1.4 cm on bone scan and remained stable on follow-up imaging. No other evidence of metastasis was identified on CT imaging of the chest, abdomen, or pelvis at the time of this publication.

## Discussion

CCPRCT, previously known as clear cell papillary RCC, was first recognized in 2006 and included in the 2016 World Health Organization (WHO) as a novel subtype.^2^ The tumor was renamed as a reflection of its almost invariably low-pathological stage and indolent behavior.^3^ Although initially described in the setting of end-stage renal disease,^4^ it is currently known to be a common tumor affecting normal and abnormal kidneys in a sporadic setting.^5,6^ Macroscopically, tumors tend to be solid or cystic, usually tan-white and fibrous.^7^ Histologically, CCPRCT shows tubular and papillary structures, as well as frequent cystic components.^7,8^ Cells characteristically have clear cytoplasm with nuclei aligned away from the basement membrane resembling secretory endometrium.^7,9^ CCPRCT classically tends to be WHO/International Society of Urological Pathology (WHO/ISUP) grades 1 or 2, with only a minority of reported tumors that have grade 3 nuclei.^3^

Immunohistochemically, CCPRCT shows cup-like staining pattern with CA9, and is typically diffusely positive for keratin 7. GATA3 can be positive while AMACR and CD10 are either negative or focally positive.^3,7^ In the areas with CCPRCT morphology, our tumor showed an immunohistochemical profile most consistent with CCPRCT. Although CD10 staining was present, it showed membranous staining in only the apical aspect of cells. Interestingly, minor labeling for CD10 has been described to have focal positivity in 24% of tumors, and when present is frequently luminal with accentuation in cells lining cystic foci as seen in our patient's tumor.^5^ In the areas with more solid nested and sarcomatoid morphology, the tumor stained similarly although showed loss of keratin 7.

Molecularly, CCPRCT does not appear to have a distinct profile and is better characterized by a lack of the characteristic genetic alterations identified in other subtypes such as CCRCC, papillary RCC, *TFE3*-rearranged RCC, or *ELOC* (formerly *TCEB1*)-mutated RCC.^10–15^ In recent years, a range of renal tumors displaying CCPRCT-like areas have been increasingly recognized and documented.^16^ Therefore, with current evidence suggesting that CCPRCTs are almost invariably indolent, the standard practice has been to use strict morphological and immunohistochemical criteria (potentially molecular studies in selected tumors) when making a diagnosis of CCPRCT, as the current terminology implies those tumors are not even malignant. When faced with a CCPRCT-like tumor showing either an unusual morphology (such as the presence of high-grade areas, tumor necrosis, or a mixture of CCRCC and CCPRCT areas), unusual immunophenotype (such as a box-like staining pattern for CA9), unusual tumor characteristics (such as advanced pathological stage), one has to assume that such tumors represent entities other than CCPRCT, with CCRCC being the most common tumor type reported to mimic CCPRCT.^16^ From a practical standpoint, if such further molecular characterization is unavailable, CCPRCT-like tumors with unusual or aggressive features likely need to be regarded and treated as an RCC.

If access is available, testing for molecular alterations seen in CCRCC, papillary RCC, or molecularly defined RCCs may be considered for CCPRCT-like tumors with high-grade morphology and advanced stage. An NGS panel for genes such as *VHL, PBRM1, SETD2, BAP1,* and *MET*, among others as well as copy number variation analysis may be helpful to help exclude both CCRCC and papillary RCC. Translocation RCCs are also well-known mimickers of many RCC subtypes, including CCRCC and CCPRCT.^17–19^ Skala et al specifically observed that *TFE3*-rearranged RCCs may have focal or diffuse subnuclear clearing and linear array of mid-to-apical nuclei on morphology akin to CCPRCT.^18^ Therefore, FISH testing for *TFE3* in addition to immunohistochemical studies for TFE3 may be considered. *ELOC* (formerly *TCEB1*)-mutated RCC are similarly known to have overlapping morphology with CCRCCs and CCPRCTs. *ELOC* mutation testing may also be considered in an NGS panel for CCPRCT-like tumors depending on available resources.^20^

Although case reports of CCPRCT-like metastatic tumors with negative genetics have been reported,^21^ our patient's tumor was unique for several reasons. First, the presence of typical CCPRCT areas, associated with the characteristic immunoprofile in both the primary and the metastatic tumors, is striking. Extraordinarily, the entirety of the metastatic deposit was CCPRCT-like. Second, the CCPRCT-like areas were notably intertwined with those with sarcomatoid morphology. Finally, we did not identify molecular evidence that would exclude a diagnosis of CCPRTC using an extensive molecular panel. More specifically, the tumor did not show any of the most commonly CCRCC mutations (eg *VHL, PBRM1, SETD2,* and *BAP1*), mutations of sarcomatoid transformation in CCRCC (eg, *NF2* and *TP53*), nor chromosome 3p loss in any of the components (primary CCPRCT-like, primary sarcomatoid, or metastatic deposit).^22–25^ As *TSC1, TSC2, MTOR*, or *ELOC* (*TCEB1*) mutations were also not identified, diagnoses such as RCC with fibromyomatous or leiomyomatous stroma and *ELOC*-mutated RCC similarly are not supported.^15,26^ The immunoprofile was also not in favor of a diagnosis of FH-deficient or SDH-deficient RCCs.^27^ Finally, immunohistochemical studies (TFE3, cathepsin-K, MART-1, HMB45) and confirmatory FISH analysis for *TFE3* did not support a *TFE3*-rearranged RCC.

The present tumor illustrates that even when using strict criteria and with extensive molecular characterization, rare cases remain perplexing and difficult to classify. Whether this tumor represents an extremely rare report of CCPRCT with sarcomatoid differentiation and metastasis, or an unclassified RCC closely mimicking CCPRCT, cannot be entirely determined. However, it clearly highlights that the entity CCPRCT versus CCPRCT-like tumors is still an evolving concept that is yet to be fully defined. Future large studies with long-term follow-up, and similar case reports with molecular characterization will be key in determining the extent to which the diagnosis of CCPRCT can be confidently made solely on morphological and immunohistochemical grounds. Ultimately, genome-scale molecular studies on similar unusual tumors would help to identify driver molecular alterations that would eventually help to refine the diagnosis or describe new unreported entities.
